# Phacomatosis cesioflammea with extracutaneous involvement in a Filipino child

**DOI:** 10.1016/j.jdcr.2026.04.044

**Published:** 2026-04-29

**Authors:** Mae N. Ramirez, Cyrene C. Tan, Marie Eleanore O. Nicolas

**Affiliations:** a Department of Dermatology, University of the Philippines – Philippine General Hospital, Manila, Philippines; b Department of Dermatology, Rizal Medical Center, Pasig City, Philippines; c Department of Dermatology, St. Luke’s Medical Center Global City, Taguig, Philippines

**Keywords:** genodermatosis, neurocutaneous syndrome, nevus cesius, nevus flammeus, phacomatosis cesioflammea, phacomatosis pigmentovascularis

## Introduction

Phacomatosis pigmentovascularis (PPV) was first described by Ota et al (1947).^1^^,^^2^ It is a rare genodermatosis, comprised of disorders presenting with vascular abnormalities and melanocytic lesions.^3^ Phacomatosis is derived from the Greek word *phakos* meaning “birthmark” or “nevus”. It applies to disorders with 2 or more different nevi and refers to the association of an extensive cutaneous vascular malformation adjacent to a dermal melanocytic nevus. Cesioflammea is derived from the Latin word *caesius* which means “blue-gray” and *flammea* which means “flame”.^4^ Most cases are sporadic with no familial tendency.

In 1985, PPV was classified into 4 types by Hasegawa and Yasuhara according to the pigmentary lesion accompanying the nevus flammeus.^1^ Each type was further subdivided depending on the existence of extracutaneous signs into *a* (cutaneous only), and *b* (with extracutaneous involvement).^5^ In 2005, Happle formulated a new classification for PPV which consists of 4 types.^6^ Torchia proposed to add phacomatosis melanorosea in 2012 and phacomatosis cesioflammeomarmorata and phacomatosis melanocesioflammea in 2021 to update Happle’s classification. The classifications are available in Table I.^7^

**Table I:** Updated classification of phacomatosis pigmentovascularis by Torchia (2021)^7^

Type	Pigmented nevus/i	Capillary nevus/i	Mosaic gene mutation
Phacomatosis cesioflammea	Nevus cesius	Nevus flammeus	GNAQ
Phacomatosis cesiomarmorata	Nevus cesius	Cutis marmorata telangiectatica congenita	GNA11
Phacomatosis spilorosea	Macular nevus spilus	Nevus roseus	PTPN11
Phacomatosis melanorosea	Flag-like hypermelanotic nevus	Nevus roseus	Unknown
Phacomatosis cesioflammeomarmorata	Nevus cesius	Nevus flammeus, cutis marmorata telangiectatica congenita	Unknown
Phacomatosis melanocesioflammea	Flag-like hypermelanotic nevus, Nevus cesius	Nevus flammeus	Unknown

A study by Fernandez-Guarino et al (2008) reported 15 cases of PPV in a 12-y period,^5^ and Shields et al (2011) reported a risk for melanoma from a study of 7 PPV patients.^8^ In 2016, there were a total of 247 cases in literature with most reported in Japan.^4^ Kumar et al in 2018 conducted a systematic review from 1982 to 2017 and found a total of 176 cases.^3^

## Case report

**Fig 1:**
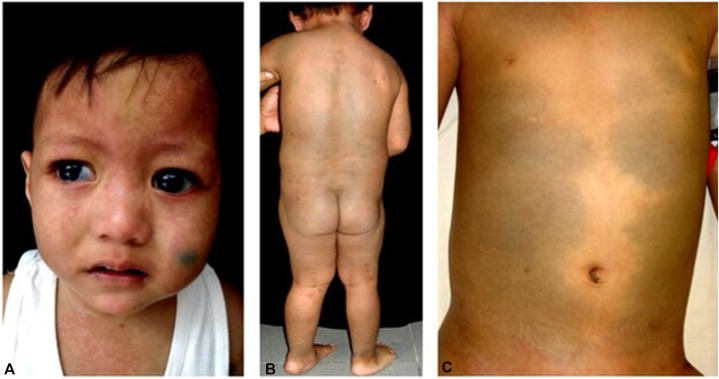
Dermatologic features of the patient with phacomatosis cesioflammea. Extensive nevus flammeus (ie, port-wine stain) predominantly on the mid-upper face (including both eyelids and forehead), with a solitary round discrete bluish patch on the left cheek **(A)**. The nevus flammeus was also present on the upper anterior chest, right palm, legs, and soles (not shown). Multiple confluent bluish-gray patches are present all over the body, most prominent on the trunk and lower extremities **(B, C)**.

We present a case of an 18-month-old male who was referred for evaluation of skin lesions. He was the fourth child of nonconsanguineous parents. There was no other remarkable heritable disorder in the family. Pregnancy was uncomplicated and the skin lesions have been present since birth. On general evaluation, the boy was healthy and alert. Skin examination revealed multiple large, dusky erythematous, ill-defined somewhat reticulate patches over both sides of the face, upper trunk, and extremities. These intermingled and overlapped with ill-defined bluish gray patches noted on the lumbosacral area, flanks, extremities, and left cheek (Fig 1). The clinical diagnosis was phacomatosis cesioflammea. Further examination noted that the left scrotum was slightly larger and the upper arm, mid-thigh, and calf circumferences were all greater on the left compared to the right. Left leg length was also noted to be greater. There were no varicosities, bruits, or thrills appreciated over the hypertrophic areas.

**Fig 2:**
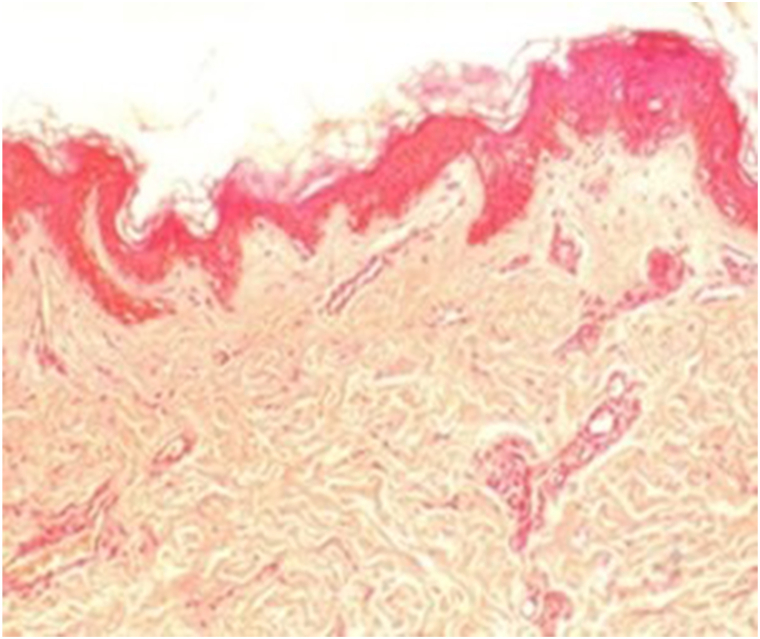
Histopathology of the port-wine stain. Dilated capillaries in the papillary and reticular dermis on hematoxylin and eosin stain, consistent with nevus flammeus.

Fig 2 depicts the histopathology of the port-wine stain.

**Fig 3:**
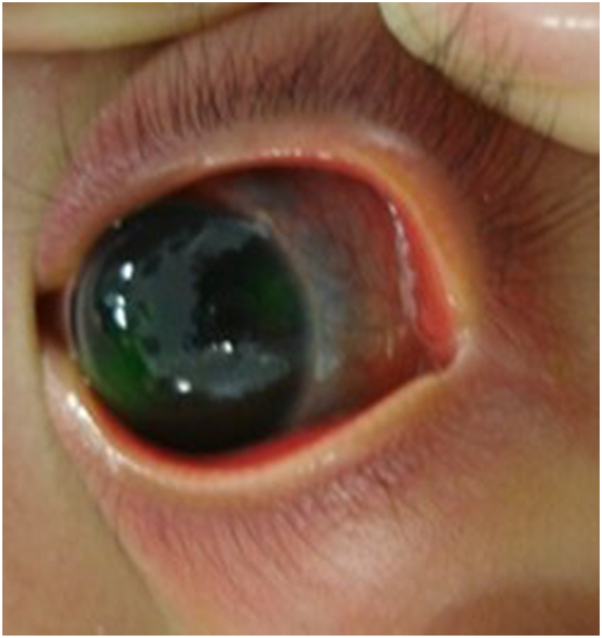
Ophthalmologic findings of the patient. Buphthalmos and bluish patchy discoloration around the limbus of the left eye (a bilateral finding in this patient).

Ophthalmologic examination revealed buphthalmos or enlargement of the eyeball, bluish patches on the sclerae, increased intraocular pressures, thinned out sclerae, increased corneal diameters, cup:disc ratio of 0.5, tilted discs, and prominent iris processes of both eyes (Fig 3) consistent with congenital glaucoma.

Skeletal survey revealed microcephaly and leg length discrepancy with the left leg length greater than the right. The hematologic, biochemical, urinary laboratory tests, chest radiograph, and whole abdominal ultrasound showed normal results. Arteriovenous Doppler ultrasound of both legs likewise revealed no venous varicosities or arteriovenous malformations. Cranial computed tomography scan and magnetic resonance imaging with contrast enhancement done to rule out cerebral calcifications, atrophy, or leptomeningeal angiomatosis showed no intracranial abnormalities. Cranial magnetic resonance angiography failed to reveal any vascular aberrations. Diffusion tensor imaging to evaluate the brain white matter was also unremarkable.

The patient underwent bilateral goniotomy with improvement of vision and decreased buphthalmos and myopia. He was referred to genetics and the multiple congenital anomalies unit for developmental screening and counseling and to rehabilitation medicine for use of compression stockings and for prevention of scoliosis that may arise from the leg length discrepancy. Genetic testing was refused because the family wished to focus on addressing the various organ systems affected by the condition.

## Discussion

The dermatologic finding in the patient’s face required differentiation of nevus flammeus from cutis marmorata. The vascular lesion on the face was never transient. Its persistence since birth, well-defined borders, unilateral distribution, and lack of a response to temperature change favored a diagnosis of the former. Moreover, the asymmetry in limbs involved both soft tissue and skeletal components as evidenced in the skeletal scan.

Other considerations for the medical condition of the patient included Sturge-Weber syndrome, which is similar to phacomatosis cesioflammea in that it is also associated with the GNAQ gene, and Klippel-Trenauney syndrome because of the presence of the port-wine stain birthmark. Although the patient had buphthalmos which could be indicative of glaucoma in Sturge-Weber syndrome, the absence of seizures, migraines, and stroke-like episodes with subsequent unilateral weakness ruled this out. Furthermore, the overgrowth of soft and skeletal tissue indeed brought up a consideration of Klippel-Trenauney syndrome, but the absence of vein and lymphatic malformations ruled this out.

In the past, PPV was believed to be explained by the genetic model of nonallelic twin spotting.^9^ Twin spots are 2 different adjacent cutaneous lesions that represent 2 different mutant cell clones resulting from a loss of genetic heterozygosity. Somatic recombination of cells heterozygous for 2 different mutations on 2 homologous chromosomes gives rise to 2 daughter cells homozygous for either mutation. Recently, phacomatosis cesioflammea has been linked to mutations in *GNA11* and *GNAQ* genes.^9^

The diagnosis of phacomatosis cesioflammea in our patient is based on the distinctive association of an extensive vascular nevus (nevus flammeus) with pigmentary lesions (aberrant Mongolian spots). About fifty percent (50%) of patients have systemic involvement. In our patient, extracutaneous manifestations were present, namely glaucoma, microcephaly, left-sided soft tissue hypertrophy, and bony hypertrophy of the left lower extremity.

Currently, there is no available consensus for the treatment of phacomatosis cesioflammea. Management is directed at identifying associations to decrease morbidity. The absence of extracutaneous associations portends a better prognosis and usually requires no treatment. Ophthalmologic and cranial nervous system management by specialists may be necessary and is recommended. Leg elevation, compression stockings, physical therapy, and rehabilitation are important in cases of leg involvement. Our patient was advised to use a shoe lift to prevent scoliosis that may arise from the leg length discrepancy. In some cases, surgical intervention may be warranted. There is rarely a need for treatment of skin lesions. Pulsed dye lasers may be used for port-wine stains. Caution must be applied in the use of Q-switched lasers for pigmentary lesions due to a high rate of recurrence and concerns about laser effects on residual pigment cells.^10^

This report sheds light on the genetic aspects of this syndrome, and stresses the importance of a multidisciplinary approach in recognizing underlying anomalies. This can guide physicians towards appropriate management, alleviating existing morbidity, and minimizing preventable complications.

## Conflicts of interest

None disclosed.
